# Immunogenetics of the Ocular Anterior Segment: Lessons from Inherited Disorders

**DOI:** 10.1155/2021/6691291

**Published:** 2021-06-28

**Authors:** Jasmine Y. Serpen, Stephen T. Armenti, Lev Prasov

**Affiliations:** ^1^Department of Ophthalmology and Visual Sciences, University of Michigan, Ann Arbor, MI 48105, USA; ^2^Case Western Reserve University School of Medicine, Cleveland, OH 44106, USA; ^3^Department of Human Genetics, University of Michigan, Ann Arbor, MI 48109, USA

## Abstract

Autoimmune and autoinflammatory diseases cause morbidity in multiple organ systems including the ocular anterior segment. Genetic disorders of the innate and adaptive immune system present an avenue to study more common inflammatory disorders and host-pathogen interactions. Many of these Mendelian disorders have ophthalmic manifestations. In this review, we highlight the ophthalmic and molecular features of disorders of the innate immune system. A comprehensive literature review was performed using PubMed and the Online Mendelian Inheritance in Man databases spanning 1973–2020 with a focus on three specific categories of genetic disorders: RIG-I-like receptors and downstream signaling, inflammasomes, and RNA processing disorders. Tissue expression, clinical associations, and animal and functional studies were reviewed for each of these genes. These genes have broad roles in cellular physiology and may be implicated in more common conditions with interferon upregulation including systemic lupus erythematosus and type 1 diabetes. This review contributes to our understanding of rare inherited conditions with ocular involvement and has implications for further characterizing the effect of perturbations in integral molecular pathways.

## 1. Introduction

Autoimmune and autoinflammatory disorders [1] are frequent causes of morbidity and mortality [2, 3] and are increasing in incidence [4, 5]. They are caused by dysregulation of various components of the innate and adaptive immune response, but their molecular pathogenesis has not been fully elucidated. The immunological continuum hypothesis [6] proposes that monogenic autoinflammatory disorders and autoimmune diseases are on a spectrum. On one end, autoinflammatory diseases represent systemic inflammation without the high-titer autoantibodies or autoreactive T lymphocytes that characterize autoimmune disorders [7]. On the other end, autoimmune diseases result from defects in the adaptive immune response and may be mediated by autoantibodies. In the middle are mixed-pattern disorders, such as psoriasis, which have dysregulation in both innate and adaptive immune responses [7]. A wide range of clinical phenotypes are associated with these disorders [2], and these include ocular phenotypes [8, 9]. Despite phenotypic variability, these disorders share many common molecular features, including perturbations in specific signaling pathways such as the phosphatidylinositol 3-kinase-protein kinase B (PI3K-Akt), toll-like receptor, and nuclear factor kappa B (NF*κ*B) pathways [10].

For autoimmune disorders, numerous susceptibility loci, particularly human leukocyte antigen (HLA) genes, have been identified since the advent of genome-wide association studies, but many causal variants in autoimmune disease remain to be discovered [11]. Similarly, autoinflammatory diseases are modulated by inflammatory cytokines including interleukins, tumor necrosis factor *α* (TNF*α*), and type I interferon. However, high-throughput sequencing has identified pathogenic variants in genetic pathways that diverge from classic cytokine regulation and inflammation. For example, *TMEM173* encodes the stimulator of interferon genes (STING) and is implicated in STING-associated vasculopathy with onset in infancy. These findings highlight the importance of studying genes beyond those involved in classic cytokine regulation and inflammation [12].

The ocular anterior segment is vulnerable to damage secondary to autoimmune and autoinflammatory processes, specifically leading to disorders of the ocular surface and corneal epithelium and stroma, as well as glaucoma [8, 9, 13–18]. These disorders can compromise vision, necessitate surgical intervention, and often lead to permanent visual impairment. While hereditary corneal dystrophies have been well-studied and reviewed [19–21], the anterior segment features of immunogenetic disorders have been less well characterized.

The ocular surface and cornea contain several defense mechanisms, including innate and adaptive immune responses. Protection of the ocular surface includes physical barriers, such as the bony orbit and eyelid, and innate immune components including cellular/molecular elements, such as the tear film, corneal epithelial cells, keratocytes, polymorphonuclear cells, and corneal nerves [22]. Langerhans cells are the antigen presenting cells (APCs) of the cornea and are part of the adaptive immune response. These cells display antigens on the cell surface using major histocompatibility complex proteins and in turn induce downstream adaptive immune response effectors [22]. Eye-derived APCs pass through the trabecular meshwork to travel in the bloodstream to the spleen [23]. Immune tolerance in the eye is also derived from anterior chamber immune deviation [24], an ocular response to inflammation that induces a regulatory T cell response via the spleen and thymus [25]. Additionally, the microenvironment of the eye is conducive to immunosuppression, with various factors expressed in the corneal endothelium and iris-ciliary body, including the B7 family of inhibitory costimulatory molecules [23].

In addition to pathways involved in ocular surface protection, recent evidence has suggested a role for the immune system in the pathogenesis of glaucoma, as animal studies have shown that T cells which have been presensitized by exposure to commensal microflora contribute to glaucomatous neurodegeneration [26]. Systemic autoimmune diseases are often associated with ocular findings, especially in rheumatoid arthritis, Sjogren syndrome, seronegative spondyloarthropathy, and antineutrophil cytoplasmic antibody (ANCA)-associated vasculitis [27]. Inflammatory conditions can affect the various layers of the eye. They manifest themselves as episcleritis, scleritis, keratitis, uveitis, and retinal vasculitis, and can cause complications such as glaucoma and corneal opacification. Ocular damage can arise secondary to treatment of autoimmune disease with agents such as hydroxychloroquine, chloroquine, corticosteroids, or bisphosphonates [27]. Monogenic autoinflammatory disorders including idiopathic granulomatous disorders, familial Mediterranean fever, tumor necrosis factor receptor-associated periodic syndrome, mevalonate kinase deficiency, and cryopyrin-associated periodic syndrome have varying ocular manifestations due to aberrant proinflammatory cytokine release, particularly IL-1*β* [28]. Additionally, keratitis, conjunctivitis, and anterior uveitis have been noted, though ocular involvement in autoinflammatory disease is heterogenous [28]. Ocular features are especially common in chronic infantile neurological cutaneous and articular (CINCA) and Blau syndrome [28].

Here, we review the evidence for how hereditary autoimmune and autoinflammatory disorders affect the ocular anterior segment. We focus primarily on Mendelian disorders of innate immune pathway components. Specifically, we describe recent advances in our understanding of four classes of genes involved in innate immunity: retinoic acid-inducible gene I (RIG-I)-like receptors, RIG-I-like downstream signaling pathways, inflammasomes, and RNA processing genes. We highlight their clinical associations with an emphasis on ocular features, human phenotypic associations, and relevant animal model/functional work. The highlighted genes are involved in RNA/DNA viral recognition and critical for host responses to exogenous viruses and other environmental triggers, i.e., ultraviolet light. Many of these genes are involved in both cellular inflammation (autoinflammation) and immune-mediated mechanisms (autoimmunity). Enriching our understanding of rare hereditary conditions will provide insight into pathway dysregulation in more common conditions, including inflammatory disorders and host-viral pathogen responses.

## 2. Methods

The RIG-I-like receptor (RLR) pathway [29] was used as a starting point to identify major genes involved in the pathway and downstream signaling. A literature review spanning 1973–2020 was conducted. Phrases that were used in PubMed database searches for the literature review included “interferonopathy,” “ocular features,” “ocular immunity,” “ectodermal dysplasia,” “Singleton–Merten syndrome,” “dyskeratosis,” “inflammasome,” “Aicardi–Goutieres syndrome,” “autoimmune,” “autoinflammatory,” “monogenic interferonopathy,” “type I interferonopathy,” “cryopyrin-associated periodic syndromes,” and “limbal stem cell deficiency.” Additional genes appearing to have implications for ocular autoimmune/autoinflammatory findings were identified from the literature. A subsequent Online Mendelian Inheritance in Man (OMIM, https://www.omim.org/) search was then performed. Papers that described clinical phenotypes and animal model work were identified in OMIM entries and reviewed. Entries for clinical conditions found to be in association with the gene of interest were searched. A subsequent complementary PubMed search for each gene and any noted clinical associations and animal model work was also performed to identify any associations that may not have been included in the OMIM database. Diagnostic criteria for conditions associated with genes of interest were inferred from OMIM descriptions and studies based on a combination of presumed pathogenic genetic variants and clinical features. Genes were classified into four subcategories: RIG-I-like receptors, RIG-I-like signaling cascade, inflammasomes, and inflammatory RNA processing disorders. To evaluate ocular and relevant tissue expression, eyeintegration v1.05 was used [30]. Pan-tissue plots were generated using the Gene 2019 dataset for the following tissues: brain (cortex), aorta, muscle, skin, whole blood, cornea-limbus, cornea-stroma, cornea-endothelium, cornea-adult tissue, cornea-fetal endothelium, cornea-cell line endothelium, cornea-stem cell endothelium, fetal and adult retina, and fetal and adult RPE.

For example, for *DDX58*, the gene was searched in OMIM (MIM: 609631). The gene-phenotype relationships section was used to identify any clinical associations. One association was noted in OMIM, Singleton–Merten syndrome 2. This entry was then selected (MIM: 616298), and the clinical features and molecular genetics sections of the entry were used to identify relevant papers describing specific clinical findings, mode of inheritance, and variant classification (loss/gain-of-function, haploinsufficiency, null). Relevant papers were accessed and additional PubMed searches were performed to ensure complete coverage of the available literature. Pan-tissue plots were generated for *DDX58* grouped with the other genes of its class (RIG-I-like receptors: *IFIH1* and *DHX58*).

## 3. Genetic Disorders and Features of Innate Immune Pathway Components

### 3.1. RIG-I-like Receptors: *IFIH1* (MDA5), *DDX58* (RIG-I), and *DHX58* (LGP2)

**Figure 1:**
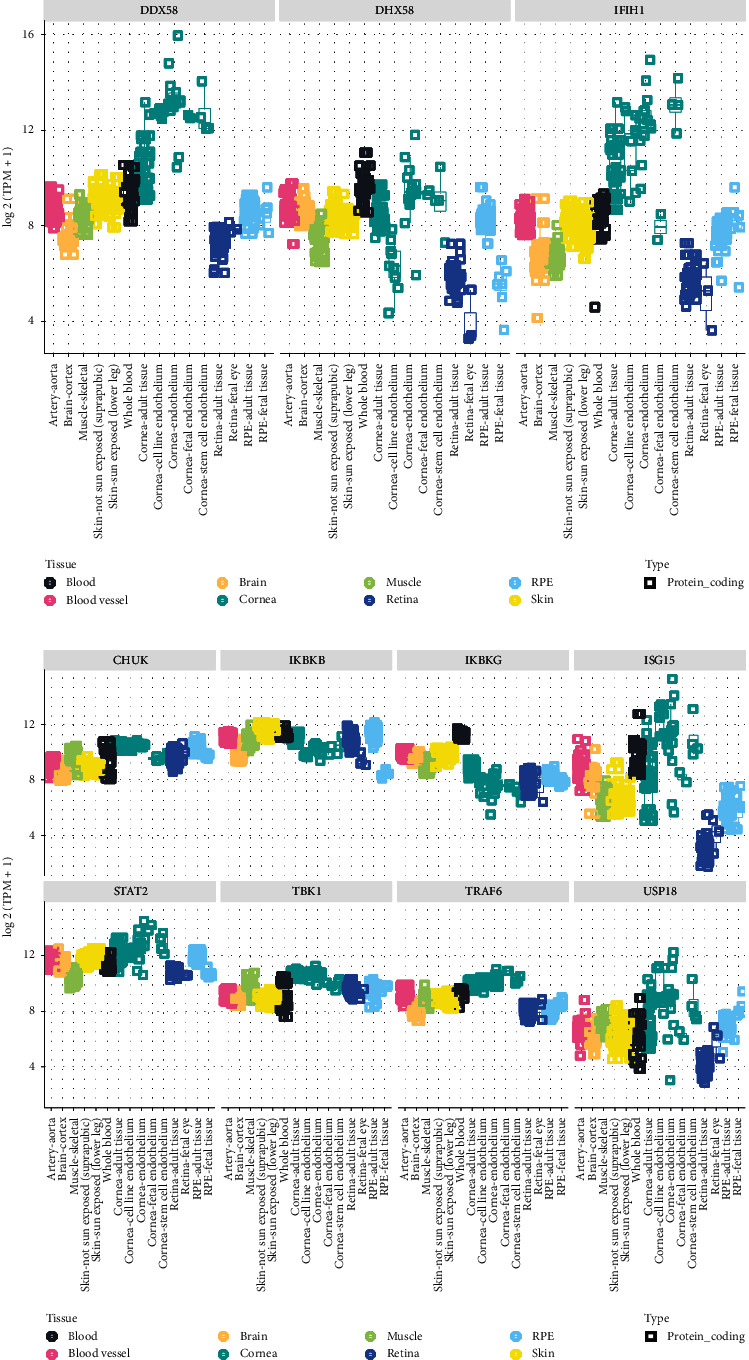
RIG-I-like receptor signaling cascade aggregated RNA-seq gene expression analyses in ocular and nonocular tissues. An eyeintegration plot showing aggregate RNA-seq data as log  2 (transcripts per million (TPM)) for the following genes: *IFIH1*, *DDX58*, *DHX58*, *TRAF6, IKKα, IKKβ, IKKγ, TBK1, STAT2, ISG15,* and *USP18*.

RIG-I-like receptors are a family of DExD/H-box RNA helicases that play a significant role in the innate immune response. They function as RNA sensors that bind exogenous double stranded RNA and activate a downstream signaling cascade that induces type I interferon signaling. The genes include *interferon-induced with helicase C domain 1* (*IFIH1*) encoding melanoma differentiation-associated protein 5 (MDA5), *DExD/H-box helicase 58* (*DDX58*) encoding retinoic acid-inducible gene I (RIG-I), and *DExH-Box Helicase 58* (*DHX58*) encoding laboratory of genetics and physiology 2 (LGP2) [31]. These receptors vary in their specificity for viral recognition. RIG-I recognizes primarily negative-strand RNA viruses with some recognition of positive-strand RNA viruses such as Zika and rubella [31–34], while MDA5 preferentially recognizes positive-strand RNA viruses, such as the picornaviruses, and binds large dsRNAs [35]. LGP2 is thought to cooperate with the other RIG-I receptors in viral recognition [36] but lacks the caspase activation and recruitment domain (CARD) involved in downstream effector signaling [37]. RIG-I-like receptors can also respond to host cellular RNAs and activate type I interferon response, likely prompting autoinflammatory conditions [38]. This may explain the activation of type I interferon pathway in response to DNA viruses such as herpes simplex virus 1 (HSV-1) [39]. All three receptors are widely expressed in ocular tissues, with the highest levels of expression in the cornea, consistent with their roles in recognizing external pathogens (Figure 1(a)).

Gain-of-function missense variants in *DDX58* (RIG-I) have been associated with Singleton–Merten syndrome type 2 (SGMRT2, MIM: 182250) [40–42], with five families reported to date. This condition is inherited in an autosomal dominant pattern with variable expressivity and is characterized by juvenile open-angle glaucoma, aortic calcification, and skeletal abnormalities (SGMRT2, MIM: 182250). Other diagnoses related to Singleton–Merten syndrome primarily include ectodermal dysplasia and Aicardi–Goutieres syndrome (AGS), which are later described. Juvenile open-angle glaucoma is the most penetrant feature of this condition, with 17/18 reported individuals having glaucoma with age at diagnosis of 2–18 years old (median 5 years old) [40–42]. The glaucoma is severe with poor visual outcomes in adult individuals and multiple incisional glaucoma surgeries required for most of the patients. The second most penetrant feature is a psoriasiform skin rash, which was present in 11/18 individuals [40–42]. Additional features include cardiac arrhythmia, valvular calcifications, joint subluxation, spontaneous tendon rupture, and various features of skeletal dysplasia and arthritis (acro-osteolysis of distal phalanx, dental anomalies including delayed secondary dentition, and short stature) [40–42]. The four reported variants *DDX58* NM_014314.3: c.1551G>C (p.Gln517His), c.1529A>T (p.Glu510Val), c.803G>T (p.Cys268Phe), and c.1118A>C (p.Glu373Ala) are found in the helicase domains of DDX58, which are involved in RNA binding. *In vitro*, these lead to basal activation of interferon responsive genes in the absence of exogenous RNA ligands or synthetic stimulation (i.e., with poly (I:C)). There is variable systemic type I interferon response; some individuals within one family show an elevated interferon signature in the blood, while others do not [41]. Recent studies suggest that there may be tissue specific activation of type I interferon response, with lesional skin showing an interferon signature and neighboring skin being unaffected [42]. It remains unclear how precisely type I interferon response correlates with disease activity in the eye. Additionally, young individuals in one family have only glaucoma, without any overt systemic features [42] suggesting the possibility of isolated ocular forms of this disorder. Gain-of-function variants show some evidence of toxicity to human trabecular meshwork cells *in vitro* supporting that the glaucoma in these patients is mediated by trabecular meshwork dysfunction [40].

Loss-of-function alleles in *DDX58* have demonstrated a key role for this gene in response to infections [43, 44]. While one mouse strain is mostly embryonic lethal in homozygotes with few animals surviving gestation, the other strain produces mice that are viable and fertile but develop inflammatory enterocolitis [43, 44]. In contrast, RIG-I overexpression in the respiratory system shows protection against influenza infection [45]. Transgenic mice carrying a bacterial artificial chromosome introducing the human *DDX58* p.Glu373Ala variant showed psoriasis-like skin lesions with IL-23/IL-17 immune axis activation and systemic inflammation [46]. Ocular features of gain or loss-of-function DDX58 mouse strains have not been examined to date.

Variants in *IFIH1* (MDA5) have been implicated in Mendelian type I interferon-associated disorders, and single-nucleotide polymorphisms (SNPs) have been associated with more common inflammatory conditions such as systemic lupus erythematosus (SLE). The clinical features of SLE and its genotype-phenotype correlations remain to be fully elucidated, and the condition can be highly variable. Thus, distinguishing some of these rare genetic conditions from lupus presents a challenge, and genetic testing may be warranted in severe, early onset forms. Multiple *de novo* and inherited dominant missense variants in *IFIH1* clustering within the two helicase domains and C-terminal domain involved in RNA recognition have been associated with Aicardi–Goutieres syndrome 7 (AGS, MIM: 615846) [47, 48]. This condition is an autosomal dominant inflammatory disorder characterized by severe neurologic impairment, with most patients presenting in infancy with delayed psychomotor development, axial hypotonia, spasticity, and brain imaging changes, including basal ganglia calcification, cerebral atrophy, and deep white matter abnormalities along with upregulated interferon signaling and interferon-stimulated gene expression (AGS, MIM: 615846). Patients with this subtype of AGS present with a variable spectrum of severity, with predominantly neurologic symptoms including developmental regression, spastic paraplegia, and microcephaly [47]. Some individuals additionally also exhibit a lupus-like syndrome with skin findings [47, 48]. However, up to 13.5% of mutation carriers may be clinically asymptomatic [47, 48]. No signs of glaucoma or anterior segment disease were reported in these individuals, but this may be related to difficulty in ascertaining these findings given the severe neurologic impairment in many of the individuals. Patients did exhibit strabismus, nystagmus, and cortical visual impairment. Other AGS-spectrum disorders (outlined below) do feature congenital glaucoma and/or optic atrophy [49]. A specific *IFIH1* gain-of-function variant in the helicase domain, *IFIH1* c.2465G>A (p.Arg822Gln), has been implicated in Singleton–Merten Syndrome type 1 [50]. These patients display variable expressivity and overlapping features with SGMRT2, with a high rate of juvenile open-angle glaucoma, along with aortic calcifications, dental anomalies, skeletal dysplasia, and spontaneous tendon rupture [50, 51]. Additionally, *IFIH1* variants have been reported in several families with primarily rheumatic disease with joint deformities and psoriatic lesions [52, 53]. When tested, *IFIH1* variants were associated with elevated interferon signature in blood and activation of type I interferon pathways *in vitro* [47, 48, 50, 52]. In addition to Mendelian disorders, *IFIH1* SNPs have been associated with type 1 diabetes (T1D), Graves' disease, and SLE [54–56], all of which can affect ocular health.

Mouse models of *IFIH1* (MDA5) gain- and loss-of-function have been generated, but ocular phenotypes have not been elucidated. *Ifih1* knockout mice are viable and fertile and show susceptibility to specific viral infections such as picornaviruses [57]. A mouse model of a gain-of-function MDA5 mutation (*Ifih1* p.Gly821Ser) was generated through mutagenesis and displayed features of lupus. The mutation was lethal in the early postnatal period in the homozygous state, and heterozygous mice developed nephritis, calcification of the liver, and infiltration in the skin, along with skeletal abnormalities [58, 59]. MDA5 overexpression mice display chronic elevations of type I interferon signaling and resistance to viral infection [60]. Ocular features in these animal models have not been reported to date.

*DHX58* encoding LGP2 is the third member of the RIG-I like receptor family, and its role in viral recognition and human disease is less clear. No variants have been associated with human disease to date or with ocular phenotypes. Mice carrying *Lgp2* knockout are viable and fertile, but ocular features have not been examined [61]. LGP2 has a context-dependent role in viral infections, acting to either inhibit or potentiate the other RIG-I-like receptors [61, 62].

### 3.2. RIG-I-like Signaling Cascade: *TRAF6*, *IKKα*, *IKKβ*, *IKKγ*, *TBK1*, *STAT2*, *ISG15*, and *USP18*

Viral replication in the cytoplasm produces uncapped RNA with a 5′-triphosphate that is recognized by RLRs [63]. Once activated in response to viral or other RNA, RLRs recruit an adaptor molecule, the mitochondrial antiviral signaling protein (MAVS), through interactions mediated by the CARD domains of RLRs [64]. Two branches of downstream signaling ensue: one is mediated by tumor necrosis factor receptor-associated factors (TRAF) 2/6 and receptor-interacting protein 1 which activate the IKK complex and nuclear factor-*κβ* (NF-*κβ*) [64]; the other activates the TANK/IKK*γ*/IKK*ε*/TBK1 complex through TRAF3, with downstream phosphorylation and dimerization of interferon regulatory factor (IRF) 3 and 7 [64]. NF-*κβ* is responsible for inducing the synthesis and secretion of inflammatory and immunoregulatory molecules [65]. The transcription factors IRF3, IRF7, and NF-*κβ* translocate to the nucleus and induce type I interferons (IFNs) [66]. They have been associated with susceptibility to infection and immunodeficiency, but no ocular associations are known (MIM: 603734, 605047, 164011). IFN*α* and IFN*β* bind to cell surface receptors and transcriptionally activate interferon-stimulated genes (ISGs) via the JAK/STAT pathway [38, 64, 67, 68]. The interferon-stimulated genes include *ISG15*, which encodes an interferon-induced protein that stabilizes ubiquitin-specific protease 18 (USP18). Of the RLR downstream signaling molecules, IKK*α*, IKK*β*, IKK*γ*, TBK1, STAT2, and ISG15 have been associated with Mendelian human disease, while TRAF6 has been explored in animal models. All genes discussed as part of this cascade are expressed in moderate levels in the cornea, with *ISG15* and *USP18* having a relatively high expression in the cornea relative to other tissues (Figure 1(b)).

IKK*α* (*CHUK*) is a catalytic subunit of the multiprotein complex IKB Kinase (IKK). It is associated with Cocoon syndrome (MIM: 613630) or fetal encasement syndrome, an autosomal recessive syndrome due to a loss-of-function truncating point mutation leading to loss of conserved helix-loop-helix ubiquitous kinase [69]. The condition is characterized by fetal malformations, including a defective face and hypoplastic limbs bound to the trunk and encased under the skin, with abnormally shiny, thick, and adhesive skin (MIM: 613630). IKK*α* is essential for skin epidermis development. Histological studies of an affected fetus demonstrated a thinned epidermis with hyperkeratosis. Ocular phenotyping revealed a spectrum of microphthalmia to anophthalmia in the affected fetuses. Null mutations of *IKKα* are associated with lethality, and fetuses with Cocoon syndrome are likely spontaneously aborted [69]. In addition to regulating epidermal and keratinocyte differentiation, IKK*α* suppresses squamous cell carcinoma which is derived from the epidermis [70]. *Ikkα* knockout mice have abnormal morphogenesis and die shortly after birth [71]. Animal model studies have established that IKK*α* is required for formation of the cornea and conjunctiva in mice; in *Ikkα* -/- eyes, the corneal epithelium and conjunctiva consist of poorly differentiated cells with round nuclei [72]. Keratinocyte differentiation and skin abnormalities were noted in IKK*α*-deficient mice [73]. Studies of mutant IKK*α* and IKK*β* in mice have established the two subunits as a link between inflammation and cancer, supporting the hypothesis that prolonged infection and inflammation confer increased risk for cancer [74].

IKK*β* is another catalytic subunit of the IKK complex. Clinically, it is associated with immunodeficiency 15A (MIM: 618204) and 15B (MIM: 615592). Immunodeficiency 15A is an autosomal dominant primary immunodeficiency disorder due to a novel heterozygous gain-of-function *de novo* variant that results in enhanced NF-k*β* signaling [75]. It is characterized by late-onset recurrent respiratory tract infections and lymphopenia, as well as immune activation of CD4+ and CD8+ T cells (MIM: 618204). Probands in two families carried heterozygous *de novo IKKβ* variants and presented with immune dysregulation, combined T and B cell deficiency, inflammation, and epithelial defects [75]. Dental enamel abnormalities consistent with ectodermal dysplasia without conical abnormalities, severe atypical eczema, and premature cataracts were among the clinical features [75]. A knockin mouse model confirmed causation. Immunodeficiency 15B is a form of severe combined immunodeficiency with autosomal recessive inheritance, caused by a homozygous truncating loss-of-function mutation [76]. It is characterized by infantile-onset life-threatening bacterial, fungal, and viral infections and failure to thrive in the setting of normal T and B cell development (MIM: 615592). No ocular features associated with immunodeficiency 15 have been described. Although IKK*β* deficiency is lethal in mice [76], in mice with epidermal-specific deletion of *Ikkβ*, severe inflammatory skin disease was noted [77].

IKK*γ* is the regulatory subunit of the IKK complex. Variants are associated with ectodermal dysplasia and immunodeficiency 1 (EDAID1), immunodeficiency 33, and incontinentia pigmenti (MIM: 300248). EDAID1 has an X-linked recessive mode of inheritance and is caused by hemizygous loss-of-function mutation (MIM: 300291). The disease is characterized by infant or early childhood onset of recurrent severe bacterial, pneumococcal, mycobacterial, and fungal infections and features of ectodermal dysplasia including conical incisors, hypo/anhidrosis, and thin skin or hair (MIM: 300291). Lymphedema and osteoporosis can also be present in severely affected individuals [78]. Immunodeficiency 33 is also X-linked recessive and is caused by hemizygous mutation that results in loss-of-function and is characterized by early onset severe infections. Patients with C-terminal zinc finger domain variants have a more severe phenotype that includes ectodermal dysplasia; variants in the leucine zipper domain or N-terminal coiled-coil domains present with milder phenotypes without ectodermal dysplasia, though the latter may have conical teeth and hypodontia [79, 80]. While other ectodermal dysplasias have been associated with dry eye [81], no ocular features have been described in association specifically with the EDAID1 subtype. Incontinentia pigmenti is an X-linked dominant condition resulting from partial gene deletion resulting in loss-of-function that manifests itself as a disturbance in skin pigmentation that is typically lethal in males; in affected females, abnormalities of the skin, hair, nails, teeth, eyes, and central nervous system can be present (MIM: 308300). In incontinentia pigmenti, ocular features are predominantly retinal (proliferative retinal vasculopathy), though microphthalmos, cataract, glaucoma, optic atrophy, strabismus, and nystagmus can occur but are likely associated with the end-stage retinopathy [82]. Ocular and dental abnormalities associated with incontinentia pigmenti were not seen in *Ikkγ* heterozygous female mice [83]. While *Ikkγ* hemizygous knockout male mice experienced lethality in utero, *Ikkγ* heterozygous females developed dermatopathy characterized by keratinocyte proliferation, skin inflammation, hyperkeratosis, and increased apoptosis [83]. Dermatologic pathology including a mutant epidermis and blocked keratinocyte differentiation [84] along with patchy skin lesions with massive granulocyte infiltration, hyperproliferation, and increased apoptosis of keratinocytes [85] has been observed in mouse models. No ocular features have been investigated in the mouse model.

*Tank-Binding Kinase 1 (TBK1)* is a kinase that activates NF-*κβ*. Activation is mediated by toll-like receptor 3, RLRs, and other cytosolic DNA sensors to induce interferon expression, and it has been established as a neuroinflammation gatekeeper [86]. Variants in this gene have been associated with susceptibility to autosomal dominant conditions including herpes-induced encephalopathy (MIM: 617900) and frontotemporal dementia and/or amyotrophic lateral sclerosis (ALS)-4 (MIM: 616439). In herpes-induced encephalopathy, the variants are missense resulting in loss-of-function [87]. With ALS-4, variants resulting in haploinsufficiency are responsible for the phenotype (with a combination of nonsense, canonical splice site, and frameshift variants) [88], and missense variants have also been shown to be enriched in ALS patients [89]. TBK1 is the binding partner of optineurin, an established glaucoma gene [90], and copy number variants in *TBK1* have been associated with normal tension glaucoma (NTG) most commonly [86, 91] as well as primary open-angle glaucoma (POAG) [90]. The duplication in association with NTG results in gain-of-function kinase activity and differs from ALS or herpes simplex encephalitis associated *TBK1* variants which cause loss-of-function or deficiency, respectively [86]. Linkage studies combined with microarray and immunohistochemistry analysis have pinpointed duplication of chromosome 12q14 as the underlying etiology for increased *TBK1* expression [92], and later studies confirmed the role of *TBK1* over flanking duplicated genes [93, 94]. The duplication has been found to be associated with NTG across multiple ethnicities [92, 95]. Triplication of *TBK1* in a large Australian cohort of NTG and POAG was found to be causal [96]. A transgenic mouse model with one extra copy of the human *TBK1* gene incorporated into the mouse genome showed that extra gene dosage of *TBK1* leads to an NTG-like phenotype similar to that seen in humans [97]. However, the mechanism of TBK1-mediated glaucoma largely remains to be elucidated.

In interferon signaling, *signal transducer and activator of transcription 2 (STAT2)* encodes a subunit of ISGF3, a transcription factor activated in the cytoplasm in response to IFN-*α* attachment to the cell wall [98]. When STAT2 is phosphorylated, it dimerizes with STAT1 and interacts with IRF9 to form a complex (ISGF3) that induces ISGs [99]. Two downstream ISGs, *ISG15* and *USP18*, negatively regulate interferon induction [100–102]. While STAT2 deficiency is associated with susceptibility to viral infection [103], monogenic type I interferonopathies result in upregulation of STAT2 activity and IFN-1 signaling [104]. Gain-of-function *STAT2* missense variants lead to type I IFN signaling, while loss-of-function variants lead to susceptibility to infection. *STAT2* variants are clinically associated with immunodeficiency 44 and a syndrome resembling pseudo-TORCH (toxoplasmosis, other agents, rubella, cytomegalovirus, and herpes simplex). Pseudo-TORCH syndrome 1 is an autosomal recessive neurologic disorder characterized by congenital microcephaly, intracranial calcifications, simplified gyration and polymicrogyria, and severe developmental delay (MIM: 251290). Pseudo-TORCH syndrome has considerable phenotypic overlap with Aicardi–Goutieres syndrome, and it has been proposed that some cases may represent the same disorder [105]. Congenital TORCH infections comprise the main differential diagnosis for Aicardi–Goutieres syndrome and can be distinguished by identification of an infectious pathogen. Serology and family history can also be useful in this process. Multigene panels can be utilized to identify pathogenic variants, though the high likelihood of identifying variants of unknown significance must be considered. Improved functional testing and characterization of variants may help to distinguish these in the future. Less common conditions included in the differential diagnosis include microcephaly-intracranial calcification syndrome, band-like calcification polymicrogyria, classic Cockayne syndrome, neonatal lupus erythematosus, Hoyeraal–Hreidarsson syndrome, mitochondrial cytopathies, 3-hydroxyisobutyric aciduria, cerebroretinal microangiopathy with calcifications and cysts, and Labrune syndrome [106]. It is important to recognize that some of these conditions likely represent phenotypic variation and share genetic etiologies. Immunodeficiency 44 is an autosomal recessive primary immunodeficiency that presents as increased susceptibility to viral infection (MIM: 616636). Patient cells are STAT2 deficient in immunodeficiency 44 [103]. An autosomal recessive condition due to *STAT2* variants that compromises the negative regulation of STAT2 by USP18, conferring gain-of-function, is characterized by immune dysregulation and neuroinflammation with infantile onset [107, 108]. A case of early onset severe autoinflammation associated with a homozygous *STAT2* gain-of-function mutation and loss of USP18 activity featured skin ulcerations, seizures, cerebral calcifications, and respiratory failure and ultimately led to death [108]. Two siblings with early onset autoinflammatory disease with neurological features and elevated IFN signature were additionally described with a homozygous missense mutation in *STAT2* resulting in gain of activity due to the inability of mutant STAT2 to interact with the STAT2-dependent negative regulator of interferon activity, USP18 [107]. Congenital cataracts have been the only associated ocular feature of pseudo-TORCH syndrome 1 to date [109]. Animal models have not been examined for ocular or skin phenotypes.

*ISG15* encodes a downstream interferon-induced protein and has been shown to stabilize USP18 [101]. USP18 is associated with autosomal recessive pseudo-TORCH syndrome 2 (MIM: 617397), which is a multisystem disorder characterized by antenatal onset of intracranial hemorrhage, calcification, brain malformations, liver dysfunction, and often thrombocytopenia (MIM: 617397). Biallelic, loss-of-function variants in *ISG15* are clinically associated with immunodeficiency 38 (MIM: 616126), an autosomal recessive condition that predisposes individuals to infection by weakly virulent mycobacteria [110]. ISG15 deficiency patients share features with various Mendelian autoinflammatory diseases associated with IFN-*α*/*β* upregulation including AGS. These include intracranial calcification and activation of ISG expression [100]. No ocular features of ISG15-associated immunodeficiency have been described, and ISG15-deficient mice do not appear to exhibit a neuroinflammatory phenotype. Unlike humans, in whom loss of ISG15's role in controlling IFN signaling does not present with unusually severe viral infection, mice are more susceptible to viral infection [111]. ISG15 has been classified as an immunomodulator in the cornea with a protective role established in fungal keratitis, suppressing inflammation via downregulation of IL-1*β* and IFN-*γ* as well as induction of fungicidal molecules CXCL10 and cathelicidin [112].

TRAF6 has no established clinical associations; however, ectodermal dysplasia has been noted in a *Traf6* -/- mouse model [113]. While the effects on the eye have not been examined, the corneal keratinocyte expression and the presence of ocular surface and corneal findings in other ectodermal dysplasias suggest that this warrants future investigation.

### 3.3. Inflammasomes: *NLRP1, NLRP3, NLRC4, AIM2,* and *AIRE*

**Figure 2:**
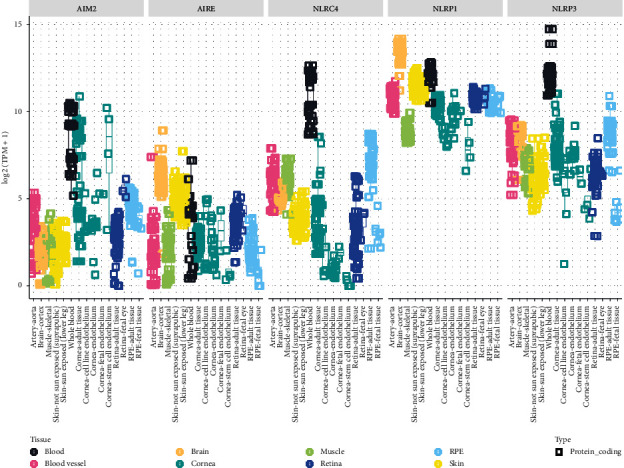
Inflammasome aggregated RNA-seq gene expression analyses in ocular and other tissues. An eyeintegration plot showing aggregate RNA-seq data as log  2 (transcripts per million (TPM)) for the following genes: *NLRP1, NLRP3, NLRC4, AIM2,* and *AIRE*.

NOD-like receptors (NLR) are an essential component of innate immunity. Inflammasomes are multimeric protein complexes in the cytoplasm that activate proinflammatory caspases and downstream cytokines including interleukin-1*β* and interleukin-18 which can lead to pyroptosis [90]. Assembly of the complex requires cytosolic pattern recognition by nucleotide binding oligomerization domain and leucine rich receptor (NLR) or absent in melanoma 2 (AIM2)-like receptor (ALR) [114]. Various inflammasomes have been identified and studied: NLRP1, NLRP2, NLRP3, NLRC4, and AIM2 [115]. Variants in inflammasome subunits are of particular interest in elucidating the genetic etiology of autoinflammatory disease. Of note, NLRP1, NLRP3, NLRC4, and AIM2 are associated with autoinflammatory diseases, enterocolitis, and cancer [116] and have been implicated in ocular disease [14]. Inflammasome activation in the eye can precipitate tissue destruction, and no protective role has been identified, whereas NLRP3 has a protective role in other tissues such as the lung [14]. All inflammasome-related genes discussed in this section are expressed in moderate levels in the cornea, with *AIM2* having a higher relative expression in corneal versus other systemic tissues (Figure 2).

NLRP1 is associated with congenital juvenile respiratory papillomatosis (MIM: 618803), vitiligo-associated multiple autoimmune disease susceptibility 1 (MIM: 606579), autoinflammation with arthritis and dyskeratosis (NAIAD) (MIM: 617388), palmoplantar carcinoma (MIM: 615225), and a new form of corneal intraepithelial dyskeratosis [117]. NLRP1 has also been implicated in acute glaucoma; in an acute IOP elevation/glaucoma mouse model (*Tlr4* -/-), it was found that TLR4 leads to increased caspase-8 expression which increases IL-1*β* expression and retinal ganglion cell death through a caspase-1 dependent pathway involving NLRP1 and NLRP3, as well as a caspase-1 independent pathway [118]. Congenital juvenile respiratory papillomatosis is an autosomal recessive condition due to missense *NLRP1* mutations resulting in gain-of-function. Clinical features include recurrent growth of papillomas on respiratory endothelial cells, and mild dermatologic abnormalities can be present [119]. *NLRP1* gain-of-function variants resulting in increased IL-1*β* secretion [120] have also been associated with susceptibility to vitiligo both in isolation and with an autoimmune and autoinflammatory disease phenotype [121]. NLRP1-associated autoinflammation with arthritis and dyskeratosis (NAIAD) is an autosomal recessive autoinflammatory disease characterized by recurrent fever, arthritis, dyskeratosis, and autoimmunity. Patients with NAIAD can present with uveitis with or without signs of corneal dyskeratosis and corneal neovascularization [122]. Palmoplantar carcinoma (corneal intraepithelial dyskeratosis and ectodermal dysplasia) is an autosomal dominant condition due to gain-of-function missense variants [123, 124]; it is characterized by keratoacanthomas in palmoplantar skin and conjunctival and corneal epithelia [124]. NLRP1 has been implicated in a novel form of corneal intraepithelial dyskeratosis, associated with a *de novo* missense mutation [117]. Keratopathy, neovascularization, corneal opacification, corneo-limbal infiltrates, limbal thickening, and keratin deposits are among the corneal findings in affected members of a French Caucasian family [117]. Palmoplantar hyperkeratosis and laryngeal dyskeratosis were also associated with the phenotype [117]. In mice, NLRP1 deficiency attenuates diabetic retinopathy [125]. Furthermore, a modest reduction in disease-free survival was observed in NLRP1-deficient mice due to a severe cutaneous inflammatory disease characterized by neutrophil infiltration of the dermis [126].

NLRP3 has been implicated in both metabolism and inflammation [127]. Gain-of-function *NLRP3* mutations are associated with cryopyrin-associated periodic syndromes (CAPS), a class of rare hereditary inflammatory disorders encompassing a continuum of phenotypes: familial cold autoinflammatory syndrome (FCAS) (MIM: 120100), Muckle–Wells syndrome (MWS) (MIM: 191900), and neonatal-onset multisystem inflammatory disease (NOMID) (MIM:607115) in order of increasing severity [128]. NLRP3 is also associated with autosomal dominant deafness 34 (with or without inflammation), postulated to be gain-of-function [129] and keratoendotheliitis fugax hereditaria [15]. Diagnostic approaches for children with suspected autoinflammatory diseases such as the cryopyrin-associated periodic syndromes have been described [130]. Developmental delay, growth restriction, and history of hospitalization for severe infections are more suggestive of immunodeficiency; night sweats, night pains, weight loss, generalized lymphadenopathy, and hepatosplenomegaly are features more consistent with malignancy. Autoinflammatory disease should be suspected in the setting of normal growth and development patterns, presence of an asymptomatic state between episodes, positive family history, and a history of similar episodes [130]. FCAS is characterized by recurrent episodes of a maculopapular rash associated with arthralgias, myalgias, fevers and chills, and swelling of extremities subsequent to cold exposure [131]. Muckle–Wells syndrome is an autosomal dominant syndrome characterized by episodic skin rash, arthralgias, and fever associated with late-onset sensorineural deafness and renal amyloidosis [132]. CINCA syndrome/NOMID is an autosomal dominant early onset severe chronic inflammatory disease characterized by cutaneous symptoms, central nervous system involvement, and arthropathy [133]. Ocular inflammation, specifically papillitis and uveitis, is a feature in severe forms of CAPS [134]. In FCAS, bilateral conjunctivitis is a feature [135], and ocular pain, photophobia, and keratitis with subsequent stromal haze have been described in a case series [18]. A relationship between MWS and bilateral conjunctivitis has also been well established [136]. Additionally, MWS can be associated with anterior uveitis, iritis, subtle corneal haze, calcific band keratopathy, optic neuritis, and papilledema secondary to hydrocephalus, glaucoma, scleritis, and episcleritis [135, 137–139]. Chronic keratitis with intrastromal epithelioid histiocytes that are also found in the skin and joints has been associated with MWS [17]. Uveitis, papilledema/optic nerve pallor, dry eye, conjunctivitis, band keratopathy, and corneal neovascularization have been noted in CINCA/NOMID [140] as well as progressive visual field defects and proptosis [133]. Keratoendotheliitis fugax hereditaria is an autosomal dominant keratitis that periodically affects the corneal endothelium and stroma, leading to central corneal opacities that can impact visual acuity [15]. A study of patients from 7 families found that patients experienced unilateral attacks of keratoendotheliitis characterized by cornea pseudoguttata and haze in the posterior corneal stroma, sometimes with a mild anterior chamber reaction; half of the patients had bilateral stromal opacities [15]. Keratoendotheliitis fugax hereditaria features a presumed autoinflammatory reaction that affects the anterior segment of the eye, and largely overlaps with the corneal manifestations of CAPS. Thus, it has been proposed that this condition is the fourth member of the NLRP3-associated periodic inflammatory syndromes [15]. A study of neurologic features in childhood-onset cryopyrin-associated periodic syndrome found neurologic involvement in half of the 12-patient cohort, including papilledema and optic atrophy; uveitis or conjunctivitis was seen in 2 children [141]. The overlap of neurologic and ophthalmic features in this syndrome highlights the importance of awareness of this rare condition across medical specialties and maintaining a broad differential inclusive of autoinflammatory disease.

Furthermore, NLRP3 is involved in the pathogenesis of acute glaucoma. Human glaucomatous eyes showed increased *NLRP3* expression along with elevation in caspase-1 and caspase-8 levels [142]. NLRP3 is upregulated in rat and mouse models of acute glaucoma [118]. Mice with a *Nlrp3* (p.Arg258Trp) mutation exhibited skin inflammation [143]. Studies of *Nlrp3* knockout mice have exhibited a protective role for NLRP3 and associated IL-18 secretion in age-related macular degeneration [144]. The anterior segment phenotype has not been examined to date.

NLRC4 is associated with FCAS 4 (described above) and autoinflammation with infantile enterocolitis (MIM: 616050) [145]. Autoinflammation with infantile enterocolitis is an autosomal dominant condition characterized by recurrent autoinflammatory flares in infancy and is caused by a *de novo* gain-of-function mutation in *NLRC4* [146]. Rodrigues et al. [145] describe two distinct clinical phenotypes for NLRC4 autoinflammatory disease: one is severe, with multisystemic inflammation beginning in the first year of life, chronic IBD, macrophage activation syndrome, or presentation suggesting cryopyrinopathy. The second phenotype is mild and defined by cold urticaria, arthralgia, ocular features (specifically conjunctivitis and dry eye syndrome), and fever [145]. Exploration of host immune response to bacterial infection has demonstrated elevated expression of the NLRC4 and NLRP3 inflammasomes in corneal ulcers of patients with infections due to *Streptococcus pneumoniae* and *Pseudomonas aeruginosa* [147]. Functional studies demonstrated that mitochondrial DNA and reactive oxygen species (ROS) are released in macrophages infected with *P. aeruginosa* [148], and mouse studies showed that type IV pilin of the bacteria can activate NLRC4 [149]. Yerramothu et al. [14] propose that ROS and type IV pilin could activate NLRC4 in corneal infections. Expression of a human *NLRC4* mutation in mice resulted in dermatitis [150], highlighting the potential for keratinocyte abnormalities in the cornea. However, the ocular phenotypes in animal models have not been examined to date.

*AIM2* is a cytosolic sensor of aberrant DNA that induces inflammasome assembly, and can recognize both foreign and self-DNA [151]. *AIM2* expression is increased in various skin conditions including psoriasis, atopic dermatitis, venous ulcers, contact dermatitis, and experimental wounds in humans. Altered expression in SLE patients has been noted. Inflammatory bowel disease, liver inflammation, brain inflammation and cell death, colorectal cancer, and autoimmune arthritis have all been linked to *AIM2* expression [151]. The specific role in ocular disease remains to be defined.

*AIRE* encodes autoimmune regulator, a transcription factor that induces expression of genes encoding tissue-restricted antigens in medullary thymic epithelial cells and is important in T cell development. Missense, nonsense, deletion, frameshift, and splice site mutations in *AIRE* are associated with autoimmune polyendocrinopathy syndrome type I (APSI), characterized by the presence of two out of three major clinical symptoms: Addison disease, hypoparathyroidism, and/or chronic mucocutaneous candidiasis (MIM: 240300). The condition also has prominent ocular findings including keratopathy and retinopathy [152]. There is no correlation between the severity of systemic disease and the ophthalmic features, and ocular involvement is heterogenous. The underlying mechanism for the ocular pathogenesis of APSI is proposed to be an autoantibody response against corneal and retinal autoantigens [152].

### 3.4. Inflammatory RNA Processing Disorders: *TREX1, RNASEH2A, RNASEH2B, RNASEH2C, SAMHD1,* and *ADAR1*

**Figure 3:**
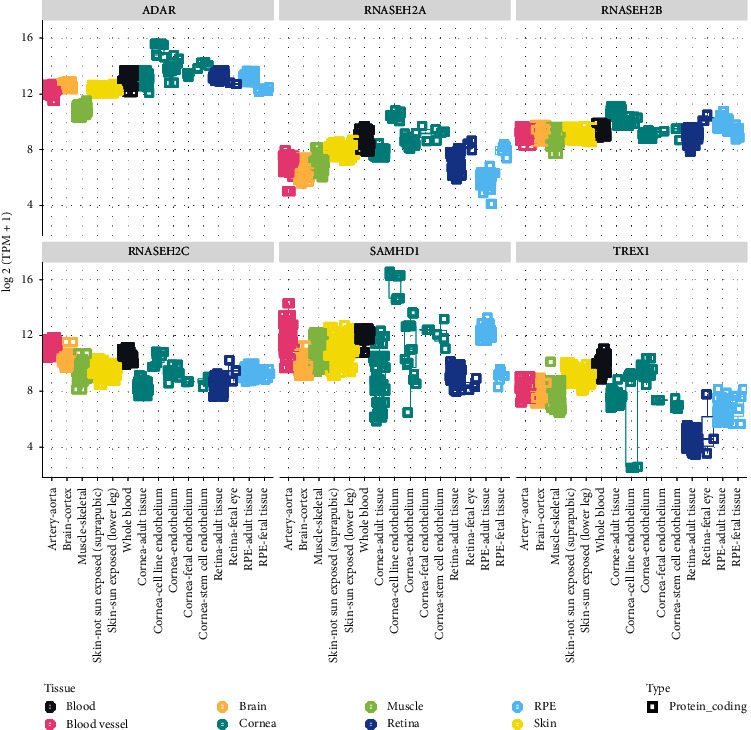
Inflammatory RNA processing aggregated RNA-seq gene expression analyses in ocular and other tissues. An eyeintegration plot showing aggregate RNA-seq data as log  2 (transcripts per million (TPM)) for the following genes: *TREX1, RNASEH2A, RNASEH2B, RNASEH2C, SAMHD1,* and *ADAR1*.

Genes involved in the inflammatory RNA processing pathways are associated with a spectrum of phenotypes. RNA processing genes are expressed ubiquitously and mostly uniformly across all tissue types (Figure 3). The mechanism of identifying foreign nucleic acids is an ancient host defense mechanism that operates in parallel to the toll-like receptor pathway to induce IFN signaling. The most common syndrome associated with defective RNA processing is Aicardi–Goutieres syndrome (AGS) (MIM: 225750), which is an inflammatory disorder characterized by microcephaly, spasticity, dystonia, and psychomotor retardation, with high risk for childhood death. There is a wide phenotypic spectrum in AGS, with some patients presenting with very early neonatal findings and others having a delayed onset later in childhood with variable degrees of neurodegeneration. In addition to the classic features described above, patients with AGS can also develop painful skin lesions, known as chilblains, and congenital glaucoma which typically presents in the first 6 months of life [49]. The genes associated with this syndrome are the *3′ repair exonuclease 1 (TREX1)*, three components of the *RNASEH2* endonuclease complex that functions on RNA/DNA hybrids *(RNASEH2A, 2B,* and *2C)*, a *Sam and HD domain-containing protein 1* that functions as a deoxynucleoside triphosphate triphosphohydrolase *(SAMHD1)*, and a protein functioning in hydrolytic deamination of adenosine to inosine in double stranded RNA *(ADAR1)*.

TREX1 (MIM: 606609) is a 3′ repair exonuclease which functions primarily on single strand DNA species. TREX1 is part of the SET complex, which is an enzymatic complex associated with the endoplasmic reticulum that localizes to the nucleus and participates in DNA degradation during certain forms of dell death. As mentioned above, not only defects in TREX1 function can lead to AGS, but mutations in this enzyme have also been shown to be associated with SLE, familial chilblain lupus (a cutaneous form of SLE), and retinal vascular and cerebral leukodystrophy (RVCL). RVCL is an adult-onset disorder characterized by central nervous system degeneration with progressive loss of vision, stroke, motor impairment, and cognitive decline, with a subset of patients exhibiting systemic vascular involvement (MIM: 192315). Variants in *TREX1* are highly penetrant in both adults and children with SLE [153, 154], suggesting that their presence confers a higher risk of developing lupus. Nine families with RVCL were found to be heterozygous for frameshift mutations in *TREX1*. These patients typically present with loss of vision due to retinal telangiectasias, microaneurysms, and retinal capillary loss [155].

Mice null for *Trex1* develop a lethal autoimmunity and die by 9 weeks [156]. This lethality is suppressed in *Trex1*-/- mice that have loss of the interferon regulatory factor *Ifn3* and the interferon receptor *Ifnar1* [157]. Expression of *Trex1* RNA can be induced by interferon-stimulatory DNA, which is a known inducer of IFN signaling in cell culture [157]. Short interspersed nuclear elements, long interspersed nuclear elements, and long terminal repeats, which are derived from endogenous retroelements, accumulate in *Trex1*-deficient mice [157] suggesting that TREX1 functions to prevent the development of autoimmunity through degrading such DNA species. There were no ocular phenotypes described in mouse models.

Interferonopathies from mutations in *TREX1* can be inherited in either an autosomal dominant or recessive fashion. By using enzyme function analysis, proteins resulting from the two dominant mutations p.(Asp200Asn) and p.(Asp18Asn) showed loss of enzyme function when the mutant protein was present in a heterodimer with the wild type protein. In contrast, heterodimers containing the recessive mutant p.(Arg114His) and normal protein did not reduce exonuclease function significantly [158].

RNASEH2 is the major type of ribonuclease H present in mammals. Its primary function is in cleaving RNA/DNA hybrids, and it is involved in DNA replication and repair. There are three subunits of the RNASEH2 complex (H2A, H2B, and H2C), all of which are associated with AGS [159]. Patients with *RNASEH2A* and *RNASEH2B* mutations have accumulation of ribonucleotides in the DNA of their cells [160, 161]. Early work pointed out that AGS due to mutations in *RNASEH2B* was due to autosomal recessive inheritance due to missense mutations. Those individuals harboring nonsense mutations were found to have missense mutations in *trans* and thus were compound heterozygotes. *RNASEH2A* and *H2C* missense variants inherited in a recessive fashion were identified in families with AGS [159].

Homozygous mice expressing RNASEH2A protein with knockin mutation p.(Gly37Ser) in the conserved catalytic domain are lethal. Quantitative reverse transcription-polymerase chain reaction displayed elevated IFN downstream gene activation without obvious neuroinflammation [162]. Similarly, *Rnaseh2b* and *Rnaseh2c* knockout mice are embryonic lethal [163, 164]. In mice with defective RNASEH2A function, increased ribonucleotide insertion in the genome was found, suggesting an essential role for RNASEH2 in genome stability. Interestingly, mice with reduction in function mutations in *Rnaseh2b* and *Ifnar1* showed a similar lethality phenotype, suggesting that systemic IFN-related inflammatory effects are unlikely the cause for embryonic lethality in *Rnaseh2* mutants [164]. Ocular phenotypes were not described in mouse models.

SAMHD1 is a 3′-exonuclease and deoxynucleotide triphosphohydrolase. Patients who present with AGS or chilblain lupus from mutations in *SAMHD1* typically present as either recessive homozygotes or compound heterozygotes [165]. Much of the function of SAMHD1 was identified in studies assessing its role in HIV pathogenesis. In cell culture, SAMHD1 can convert deoxynucleotide triphosphates to deoxynucleoside and triphosphate. In this way, it is thought to inhibit retroviral reverse transcription [166]. SAMHD1 can also function to prevent release of inflammatory ssDNA elements from replication forks [167]. This protein was first identified in a cell culture model, where mouse macrophages transfected with immunostimulatory DNA were shown to induce *Samhd1* expression. This expression was abrogated in SAMHD1-deficient mice that were *Ifnar1* deficient [165]. Mice null for *Samhd1* do not have a clinical autoimmune disease but do have elevated IFN levels in multiple tissues [168, 169], and ocular phenotypes were not described.

ADAR1 is RNA-specific adenosine deaminase involved in RNA editing which destabilizes dsRNA by converting adenosine to inosine in dsRNA. In addition to AGS, patients heterozygous for mutations in *ADAR1* can present with dyschromatosis symmetrica hereditaria, which is typically a benign condition where patients present with variably pigmented macules on the skin [170]. Patients with AGS due to ADAR1 typically do not present with glaucoma [111]. Mice homozygous for a catalytically inactive mutation in *Adar1* p.(Glu861Ala) are embryonic lethal at day 13.5 and have elevated IFN signature [171, 172]. This effect can be suppressed in MAVS-deficient mice. As described above, MAVS is a downstream effector of the RIG-1-like receptor MDA5 encoded by *IFIH1* [173] and is essential for the innate immune response. Interestingly, loss-of-function of ADAR1 in tumor cells greatly sensitizes cells to destruction by immunotherapeutic agents such as PD1 inhibitors. Bypassing such an inhibitory checkpoint may allow for effective application of such agents in certain tumor genotypes [174].

## 4. Conclusions

**Figure 4:**
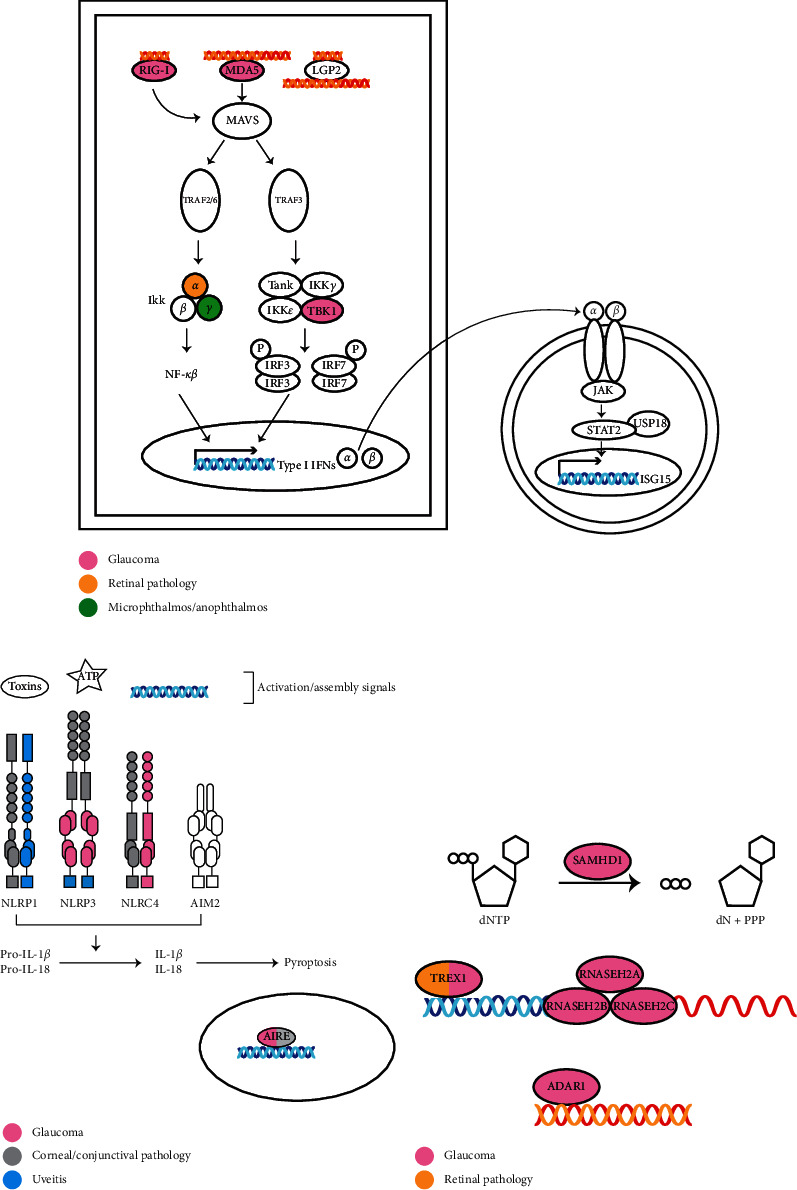
Schematic diagram of various ocular phenotypes in Mendelian disorders of innate immune pathway components including the RIG-I-like receptor signaling pathway, inflammasomes, and RNA processing enzymes. (a) The RIG-I-like receptor signaling pathway. Each receptor recognizes dsRNA of various lengths. RIG-I and MDA5 receptors signal downstream through the mitochondrial antiviral signaling complex (MAVS). Downstream effects are triggered by two pathways, leading to the ultimate translocation of transcription factors NF-*κβ* and IRF3/7 into the nucleus to induce transcription of type I IFNs, which bind to cellular receptors and induce interferon-stimulated gene transcription via the JAK/STAT pathway. Associations of individual genes with glaucoma, retinal pathology, and micro/anophthalmos are depicted. (b) Inflammasome complexes are assembled in response to activating signals and active IL-1*β* and IL-18 leading to pyroptosis. AIRE is a nuclear transcriptional regulator. Associations with glaucoma, corneal/conjunctival pathology, and uveitis are depicted. (c) RNA processing enzymes that process dNTPs, dsDNA, DNA/RNA complexes, and dsRNA have been found in association with glaucoma.

The ocular surface plays a key role as a physical and immunologic barrier to prevent infection. In addition, the eye is a relatively immune privileged site. Both the immune privilege and infection barrier can be compromised in the setting of inflammation. For instance, the higher relative expression of *DDX58, IFIH1, STAT2, ISG15, USP18,* and *AIM2* in the cornea relative to other tissues (Figures 1(a) and 1(b)) suggests a potential role for the cornea in mediating ocular inflammation. Based on our review, we have identified several genes that we expect to be associated with ocular disease (Figure 4). The lack of identified associations between variation in these genes and ocular and systemic disease may be twofold. First, these genes have extensive roles in cellular physiology, and variation may not be tolerated or compatible with life. Second, these associations may have yet to be discovered. The genes highlighted in this review have broad roles in DNA/RNA recognition and response. Understanding how such variants impact the immune response has implications beyond the context of the diseases discussed in this review. In fact, dysregulation of the interferon response has been implicated in the pathogenesis of diseases such as COVID-19 [175].

The ocular phenotypes of specific conditions discussed in this review suggest a role for screening for ocular findings in conditions such as SGMRT, AGS, and ectodermal dysplasia. There is considerable phenotypic overlap between the syndromic conditions described, suggesting common disease pathogenesis with overlapping effects on eye structures (Figure 4). For example, dental abnormalities feature prominently across SGMRT, ectodermal dysplasia, and immunodeficiency 15A. Likewise, pseudo-TORCH syndrome has considerable phenotypic overlap with AGS, and it has been proposed that some cases may represent the same disorder [105]. As glaucoma is a well-defined feature of SGMRT, it may also be an undetected feature in AGS patients that has not been possible to characterize due to severe neurologic impairment. *IFIH1*, involved in the pathogenesis of AGS, has enriched corneal expression relative to other tissues, supporting a role in the anterior segment.

The insight drawn from the study of ocular manifestations of rare diseases has implications for common conditions such as SLE and glaucoma. The mechanism of *TBK1*-mediated glaucoma largely remains to be elucidated. However, its central role in RIG-I signal transduction suggests that *TBK1* could represent an important therapeutic target. A TBK1 inhibitor (compound II) was found to inhibit interferon response and improve autoimmune phenotypes of *Trex1* knockout mice [176]. Further study of glaucoma in SGMRT may uncover a link between glaucoma and infection or inflammation, and a role for IFN signaling in more common forms of glaucoma. As *IFIH1* SNPs are associated with common conditions like SLE, in which type I IFN is elevated, it is possible that these pathways are active in the eye in SLE and contribute to ocular disease. Type I IFN has also been implicated in the pathogenesis of T1D, for which genetic risk loci have been identified in genes that modulate the response to type I IFN [177]. Type I IFN has an influence on the beta islet microenvironment through potential mediation of the interaction between cytotoxic T cells and pancreatic beta cells. Additionally, monogenic interferonopathies may be good candidates for immunotherapy directed toward blocking IFN signaling, and these may be better targeted therapies for the corneal disease and glaucoma associated with these conditions. Furthermore, populations with elevated IFN may be at high risk for conditions that are associated with AGS and SGMRT, such as glaucoma, and may warrant more frequent surveillance.

The utility of surgical intervention for glaucoma and other ocular conditions in the setting of autoimmune/autoinflammatory disease is also worthy of consideration, as the risk of procedural failure and infection is higher for patients who have immunologic disorders. Corneal grafts transplanted in eyes not treated with adjuvant immunosuppressive therapy failed in 50% of cases [178]. In these immunogenetic conditions, immune privilege may be compromised leading to graft rejection by similar mechanisms as in solid organ transplants [179]; therefore, these cases may require a different immunosuppression approach from standard corneal transplantation cases. The various inflammatory mediators and effectors in type I interferonopathies may serve as potential drug targets. It is possible that immunomodulatory therapy in the setting of corneal or glaucoma surgery may improve surgical outcomes and disease control. Potential treatments of type I interferonopathies vary based on pathogenic mechanisms. Strategies include blocking the generation and sensing or signaling of nucleic acids, as well as targeting the interferons themselves with antibodies [104].

The limitations of this study are largely diagnostic in nature, as the causal relationships between the genetic variants and the aforementioned diseases remain to be established in most cases. The ability to interpret the variants discussed is dependent on functional studies that remain to be done, given the high level of natural variation in some of the genes included in this review. Establishing pathogenicity could be done by evaluating interferon signature in skin lesions from patients, for example, [42]. In the absence of familial segregation or clear functional pathogenesis, most variants remain classified as variants of unknown significance. High-throughput functional evaluation of pathogenesis is thus a critical area for future research efforts.

This review highlights the current knowledge about a subset of monogenic innate immune disorders affecting the ocular anterior segment. Despite the many advances in understanding innate immunity and the eye, many of the genes in these pathways have not been implicated in human disease or studied in the eye. Even among genes with established pathogenesis in Mendelian type I interferonopathies, there is significant variation in clinical presentation and overlap between clinical findings. This review has implications for further functional analysis and clinical genetic testing, as it is likely that milder variants in the genes in this review are contributing to other diseases that have not been discovered or milder forms of more common diseases such as SLE. Additionally, for mixed-feature autoinflammatory/autoimmune disorders like psoriasis, genes like *DDX58* and *IFIH1*, which are correlated with clinical conditions that include psoriatic features, may offer insight into the pathogenesis of psoriasis and other mixed-feature conditions along the immunologic continuum. The implication of the RIG-I-like receptor signaling, inflammasome, and RNA processing pathways in more common disorders like SLE and glaucoma also warrants further investigation.

## Data Availability

All relevant data are included within the manuscript text and figures.
